# Audit on the Recommendation of Exercise by Clinicians in a Community Mental Health Team

**DOI:** 10.1192/bjo.2025.10554

**Published:** 2025-06-20

**Authors:** Cissy Atwine, Joe Parvin, Sally Axelby, Isabelle Akin-Ojo

**Affiliations:** Sussex Partnership NHS Foundation Trust, Horsham, United Kingdom

## Abstract

**Aims:** Determine the proportion of patients who have exercise documented as part of their initial treatment plan. Identify any trends in exercise recommendations across different mental health conditions. Highlight areas for improvement and develop strategies to increase the incorporation of exercise recommendations in clinical practice.

**Methods:** A retrospective review of patient records from September to October was conducted, focusing on documentation of exercise recommendations in their initial assessment. Data extracted included patient demographics, diagnoses, documentation of exercise advice, type of exercise (if specified), and clinician type. Variations by diagnosis and clinician practice were analysed.

**Results:** A total of 63 patient records were reviewed and of these only 4 (6.3%) had a documented recommendation of exercise in their care plan. Of these, 2 patients were recommended to go for walks, one to continue with horse riding, there was no specific recommendation noted for the fourth patient.

26 (41%) of the patients had a diagnosis of depression, 16 (25%) of anxiety, 11 (17%) had ADHD and for 12 (19%) patients, there was no diagnosis recorded. 27 (43%) of patients had pre-existing physical co-morbidities. 25 (40%) were seen by a Doctor, 24 (38%) by a Mental Health Wellbeing Practitioner and 14 (22%) by a Lead practitioner (Mental Health nurse or social worker).

**Conclusion:** The audit highlights a gap in the integration of exercise recommendations into care plans, despite strong evidence supporting its benefits for mental health, in particular for depression and ADHD. A re-audit will be done in 3 months’ time to assess progress after presenting our findings to the team, creating aide-mémoire and relevant resources for staff and patients/carers.

